# The Value of Lung Ultrasound Score in Neonatology

**DOI:** 10.3389/fped.2022.791664

**Published:** 2022-05-11

**Authors:** Haifeng Zong, Zhifeng Huang, Jie Zhao, Bingchun Lin, Yongping Fu, Yanqing Lin, Peng Huang, Hongyan Sun, Chuanzhong Yang

**Affiliations:** Neonatal Intensive Care Unit, Affiliated Shenzhen Maternity & Child Healthcare Hospital, Southern Medical University, Shenzhen, China

**Keywords:** neonate, lung ultrasound (LUS), point of care, quantitative score, neonatal intensive care unit (NICU)

## Abstract

Point-of-care lung ultrasound (LUS) is increasingly applied in the neonatal intensive care unit (NICU). Diagnostic applications for LUS in the NICU contain the diagnosis of many common neonatal pulmonary diseases (such as Respiratory distress syndrome, Transient tachypnea of the newborn, Meconium aspiration syndrome, Pneumonia, Pneumothorax, and Pleural effusion) which have been validated. In addition to being employed as a diagnostic tool in the classical sense of the term, recent studies have shown that the number and type of artifacts are associated with lung aeration. Based on this theory, over the last few years, LUS has also been used as a semi-quantitative method or as a “functional” tool. Scores have been proposed to monitor the progress of neonatal lung diseases and to decide whether or not to perform a specific treatment. The semi-quantitative LUS scores (LUSs) have been developed to predict the demand for surfactant therapy, the need of respiratory support and the progress of bronchopulmonary dysplasia. Given their ease of use, accuracy and lack of invasiveness, the use of LUSs is increasing in clinical practice. Therefore, this manuscript will review the application of LUSs in neonatal lung diseases.

## Introduction

Lung ultrasound (LUS) has been increasingly used for the assessment of neonatal illness, and a LUS guideline has been proposed to facilitate standardization of this tool (1). Owing to the small chest size and the absence of obesity and heavy musculature, LUS easily and quickly recognizes the neonatal lung. LUS is performed at the bedside and can be performed quickly, facilitating prompt diagnosis and intervention and providing real-time information on pulmonary diseases. Studies have demonstrated high interobserver agreement among clinicians trained in LUS (2, 3). Moreover, LUS is known to have a steep learning curve and is easy to learn (4). LUS significantly improves the diagnosis and differential diagnosis of various neonatal respiratory diseases (5, 6). Importantly, chest X-ray (CXR) examinations in the NICU were significantly reduced. Additionally, LUS is known to have a higher diagnostic accuracy than CXR (7–9).

**Figure 1:**
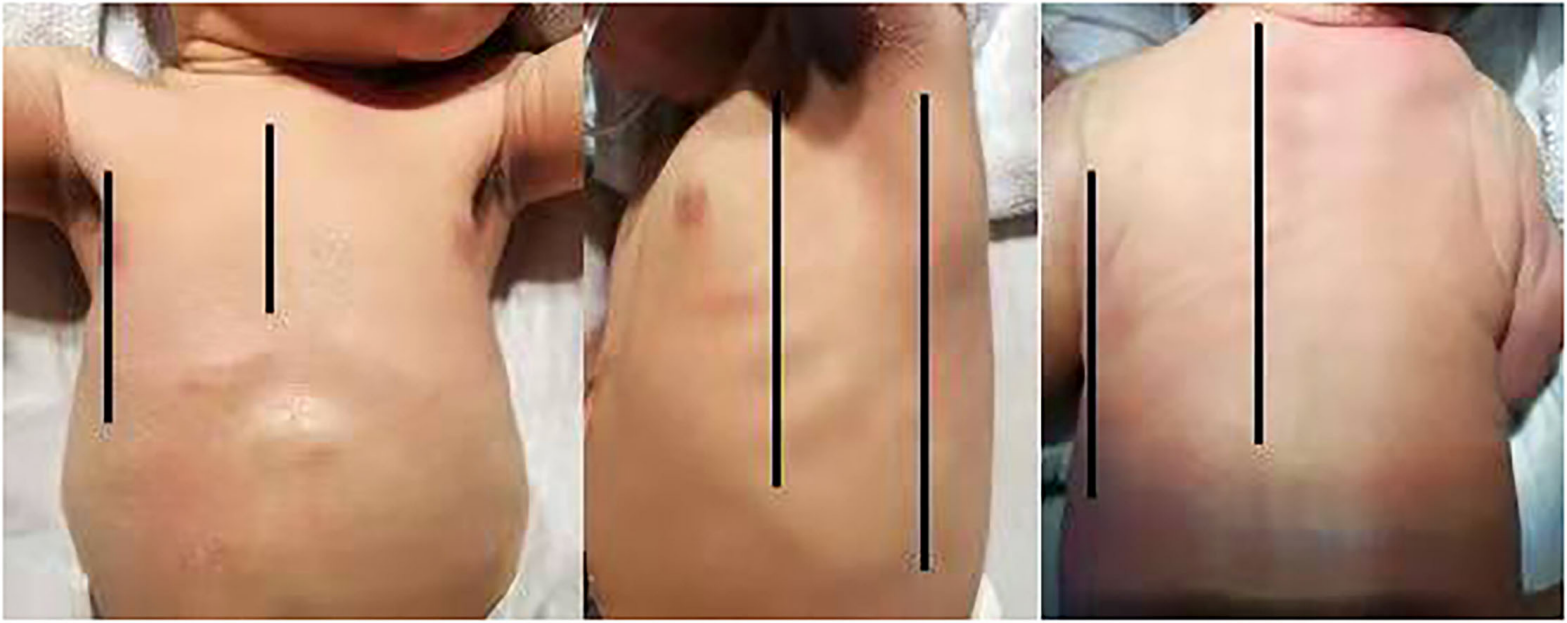
The chest surface was divided into three regions by the anterior and posterior axillary lines as boundaries: anterior region (from parasternal to anterior axillary line), lateral region (from anterior to posterior axillary line), and posterior region (from posterior axillary to paravertebral line).

It is extensively known that qualitative LUS has been adopted for diagnosing common neonatal pulmonary diseases. With the development of LUS, semi-quantitative scoring systems have since been developed to assess lung aeration dynamically and guide clinical therapy. LUS has now moved on from qualitative diagnosis, spreading to semi-quantitative evaluation of lung illness. To calculate a pulmonary ultrasound score, the chest is first divided into different areas, and to standardize the scanning protocol, the chest surface was divided into three regions by the anterior and posterior axillary lines as boundaries (Figure 1): anterior region (from parasternal to anterior axillary line;), lateral region (from anterior to posterior axillary line) and posterior region (from posterior axillary to paravertebral line). Subsequently, a number is assigned for each area according to the ultrasound findings. The scores are mainly based on the number of B lines and/or sub-pleural consolidation. In addition, this variable is based on the type of score used. The sum of the scores of all areas provides an overall score, allowing the assessment of the severity of the neonatal lung disease in a given subject. Moreover, it allows objective comparisons with other infants. The neonatal score was first described by Brat et al. (3). Each lung has been divided into three areas (upper anterior, lower anterior, and lateral) and a score from 0 to 3 for each area: 0 defined by the presence of the only A-lines; (1), defined as the presence of ≥3 well-spaced B-lines; (2), defined as the presence of crowded and coalescent B-lines with or without consolidations limited to the subpleural space; and (3), extended consolidations. The sum of the individual scores represents the infant's overall score. Therefore, score has values from 0 (completely normal) to 18. At present, most researches refer to Brat's partitioning and scoring system while some people have also proposed different strategies (Table 1). This manuscript will review the application of LUSs in neonatal lung diseases.

**Table 1:** Summary of included studies of LUSs evaluating neonatal lung diseases.

**Study**	**LUS areas**	**LUS scan**	**LUS score**	**LUS equipment**
Brat et al. (3)	Three areas in each lung (upperanterior, loweranterior, and lateral)	Both transverse and longitudinal scans. Patterns were photographed during a longitudinal scan.	A 0- to 3-point score was given: 0, defined by the presence of the only A-lines; 1, defined as the presence of ≥3 well-spaced B-lines; 2, defined as the presence of crowded and coalescent B-Lines with or without consolidations limited to the subpleural space; and 3, extended consolidations	A linear probe (12-18MHz; GELogiqE9; GEHealthcare).
Perri et al. (9)	As per Brat et al. (3)	As per Brat et al. (3)	0, only A-lines; 1, A-lines in the upper part of the lung and coalescent B-lines in the lower part of the lung or at least 3 B-lines; 2, crowded and coalescent B lines with or without consolidations limited to sub-pleural space; 3, extended consolidation	A linear probe (12 MHz, a LOGIQ E9 General Electrics ultrasound machine).
Raimondi (10)	As per Brat et al. (3)	As per Brat et al. (3)	As per Brat et al. (3)	A linear or microlinear probe (10–15 MHz)
De Martino et al. (11)	As per Brat et al. (3)	As per Brat et al. (3)	As per Brat et al. (3)	Microlinear hockey stick probe (15 MHz, CX50; Philips Healthcare, Eindhoven, Netherlands).
Perri et al. (12)	As per Brat et al. (3)	As per Brat et al. (3)	As per Perri et al. (9)	As per Perri et al. (9)
Pang et al. (13)	Six areas in each lung (upper and lower areas of anterior, posterior, and lateral sections).	As per Brat et al. (3)	As per Brat et al. (3)	A linear probe (>7.5 MHz, Voluson S8, GE Healthcare, Waukesha, WI, USA)
Raschetti et al. (14)	As per Brat et al. (3)	As per Brat et al. (3)	As per Brat et al. (3)	A micro-linear, hockey stick probe (15 MHz, CX50; Philips Healthcare, Eindhoven, The Netherlands)
Rodriguez-Fanjul et al. (15)	Three areas in each lung (anterior, lateral and posterior)	Longitudinal scans	As per Brat et al. (3)	A linear probe (12 MHz, Sonosite Edge II)
Gregorio-Hernández et al. (16)	As per Brat et al. (3)	As per Brat et al. (3)	A: A-lines: normal lung aireation, normal pleural line (=1 point). B: B-lines: vertically oriented artifacts indicating interstitial syndrome, they erase A-lines (If ≥ 3=2 points). C: White lung: multiple and coalescent B-lines with thickened pleural line, severe interstitial syndrome, with or without small subpleural consolidations (3 points)	A high-frequency hockey-stick probe (15 MHz, Philips CX50 ultrasound scanner)
Vardar et al. (17)	As per Brat et al. (3)	Longitudinal and transverse scan	As per Brat et al. (3)	A linear probe (≥7.5MHz)
Alonso-Ojembarrena et al. (18)	As per Brat et al. (3)	Longitudinal scans	As per Brat et al. (3)	A linear probe (8–15 MHz, Sonoscape Medical Corp., Shenzhen, China)
Abdelmawala et al. (19)	As per Brat et al. (3)	As per Brat et al. (3)	A 0- to 3-point score was given: (1) 0, normal lung aeration; (2) 1, separated B lines;. (3) 2, Coalescent B lines and thick pleura; (4) 3, the same as (3) with subpleural air bronchogram	L14 linear transducer (Zonare Ultrasound-SP; Mountain view, CA).
Oulego-Erroz et al. (20)	Four zones in each lung (upper anterior, lower anterolateral, lower posterolateral, and lower posterior)	As per Brat et al. (3)	As per Brat et al. (3).	A linear-array probe (L25x, Sonosite, Fujifilm Japan)
Loi B et al. (21).	As per Brat et al. Additionally, an extended score (5 per side) including the upper posterior and lower posterior areas	As per Brat et al. (3)	As per Brat et al. (3)	A hockey stick micro-linear (15 MHz);a broadband linear (10 MHz) probe
Aldecoa-Bilbao et al. (22).	Three areas in each lung (mid-clavicular line, anterior axillary line, and posterior axillary line).	Longitudinal orientation	As per Brat et al. (3)	A linear probe (13-5MHz, Siemens Acuson X300)
Liu et al. (23).	6-region (upper anterior, lower anterior, and lateral), 10-region (upper anterior, lower anterior, lateral, and upper posterior and lower posterior), and 12-region (upper anterior, lower anterior, upper lateral, lower lateral, upper posterior, and lower posterior).	Both transverse and longitudinal scans.	As per Brat et al. (3)	A linear probe (9.0 MHz, M7 Series, Mindray)
Alonso-Ojembarrena et al. (24)	As per Brat et al. Additionally, posterior field was also added.	Longitudinal scans	As per Brat et al. (3)	A linear probe (8–15 MHz Sonoscape Medical Corp., Shenzhen, China)
Szymański et al. (25)	Four areas: anterior (left), anterior (right), posterior (left) and posterior (right)	Transversal and longitudinal scans.	Five-grade scale, 0, Normal lung; 1, B lines; 2, “White lung”; 3, “White lung” and fluid alveologram; 4, “White lung” and consolidations	A linear probe (12–5 MHz, Phillips HD 11 scanner)
Eltomey et al. (26)	Six areas in each lung Each hemithorax (upper anterior, lower anterior, upper lateral, lower lateral, upper posterior, and lower posterior)	Transversal scans.	A 0- to 3-point score was given: 0, normal aeration; 1, ≥3 separated B-lines; 2, coalescent B-lines or curtain sign; 3, lung consolidation was present	A linear probe (6–12MHz, the Siemens Acuson X300 ultrasound machine, Germany)
El Amrousy et al. (27)	As per Brat et al. (3)	As per Brat et al. (3)	As per Brat et al. (3)	A linear probe (12-MHz, SIEMENS ACUSON X300)

## The LUSs in Neonatal Respiratory Distress Syndrome

Neonatal respiratory distress syndrome (RDS) is characterized by respiratory distress (RD) exacerbated progressively after birth. It is a common condition that mainly affects premature neonates and is caused by surfactant deficiency and dysfunction, which results in alveolar collapse and decreased lung aeration (28, 29). A certain degree of surfactant damage is possible in severe or long-lasting transient tachypnea of newborn (TTN) (30). Extremely preterm neonates benefit the most from optimized and timely surfactant administration because they are at a higher risk of long-term respiratory sequelae and may require repeated surfactant treatment (30–32). Early surfactant administration within the first 2 h of life reduces the risk of death, air leaks, and bronchopulmonary dysplasia (BPD) (33).

Lung aeration can usually be assessed using CXR, which provides a static assessment of lung aeration and therefore cannot be used to monitor lung recruitment during interventions. Repeated CXR exams are largely limited by the negative effects of ionizing radiation, to which neonates are thought to be more susceptible (34). However, the LUS guideline is already available to diagnose neonatal lung diseases (1). LUS is highly sensitive and specific for the diagnosis of RDS, performing better than CXR. A systematic review found LUS to have a sensitivity and specificity of 97 and 91%, respectively, which were higher than the clinical diagnosis or CXR results (7).

In addition to being used as a diagnostic tool, LUSs has been proposed to monitor the progress of disease, to decide whether to perform a specific treatment, assess the severity of RDS, and evaluate the prognosis. LUSs is a non-invasive technique with a significant link to oxygenation status and prediction of non-invasive ventilation failure (3, 35). A multicenter study provided evidence that a significant correlation between LUSs and the oxygen saturation/inspired oxygen ratio persists during the infant's NICU stay (10).

According to European guidelines, surfactant replacement should be performed when oxygen requirements are increasing and when FiO_2_ > 0.30 on CPAP pressure of at least 6 cm H_2_O (36). However, arbitrary thresholds of FiO_2_ might not accurately reveal the oxygenation status, and FiO_2_ requirements may be slow to increase, thus delaying surfactant administration after the best time frame for optimal efficacy. The predictive utility of the LUSs regarding the need for surfactant therapy is linked to the earlier presence of ultrasound findings typical of RDS compared to detectable clinical features. This would make it possible to carry out therapy with exogenous surfactant in newborns who will later develop the clinical features required by current treatment guidelines (36). In 2015, Brat et al. published the first study to test the ability of LUSs to predict the need for exogenous surfactant therapy in neonates with RDS (3). In this study, 130 neonates were enrolled, and each lung was divided into three areas (upper anterior, lower anterior, and lateral). For each lung area, a 0–3 point score was given. LUS was performed as soon as possible after admission to the NICU and before administration of surfactant. Surfactant protocol was based on European guidelines. Significant correlations were found between LUSs and oxygenation indices, indicating lung aeration. The authors found that LUSs performed better in more preterm infants. In infants with gestational age (GA) <34 weeks, the area under the curve (AUC) was 0.93 with a 95% confidence interval (CI) of 0.86–0.99 and in infants with GA ≥34 weeks, the AUC was 0.71 (95% CI: 0.54–0.90). The LUSs showed good reliability to predict surfactant administration in preterm babies with GA <34 weeks who were treated with continuous positive airway pressure (CPAP) from birth. The LUSs help to correctly identify babies who need exogenous surfactant therapy and give them surfactant as early as possible without waiting for further oxygenation worsening. Similarly, De Martino et al. published a prospective study on the predictive accuracy of LUS regarding the need for exogenous surfactant therapy in infants with GA ≤30 weeks (11). Scans of the anterior and lateral chest walls were performed. The result suggested that LUSs is significantly correlated with the oxygenation index even after adjustment for GA. The AUC in the global population was 0.94 (95% CI: 0.90–0.98), with excellent performance in both subgroups of infants with GA > or ≤28 weeks, with values of 0.98 and 0.93, respectively. However, the need for a second dose of surfactant was predicted with less accuracy, with an AUC of 0.80 (95% CI: 0.72–0.89). Perri et al. compared LUSs and CXR scores to predict surfactant administration early in newborns with RDS in a prospective study (9), and 56 newborns with a mean GA of 31 weeks were enrolled. LUSs showed a higher AUC than X-ray scores in the early recognition of infants with RDS requiring surfactant treatment. Along this line, the author also explored the change in LUSs 2 h after surfactant administration (12). They found that LUSs 2 h after surfactant administration can be useful in identifying patients who will need a second treatment. In particular, a score ≥7 showed a sensitivity of 94% and a specificity of 60%. Pang et al. scanned 12 areas (upper and lower areas of anterior, posterior, and lateral sections) of neonates with RDS and found that the LUSs increased with RDS severity (13). The LUSs for RDS vs. non-RDS showed 80.2% sensitivity and 100% specificity using a cut-off of 21.5 (Area under the ROC curve, AUC = 0.938; *P* < 0.001). The LUSs for severe vs. mild/moderate RDS showed 73.1% sensitivity and 95.7% specificity using a cut-off of 25.5 (AUC = 0.944; *P* < 0.001). Based on these results, a quality improvement project on echography-guided surfactant THERapy (ESTHER) was recently carried out by Raschetti et al. (14). They found that the ESTHER method increased the number of neonates receiving surfactant within the first 3 h of life, reduced the peak FiO_2_ before surfactant replacement, decreased the duration of invasive ventilation, and increased ventilator-free days. The global need for surfactant did not significantly change. Rodriguez-Fanjul et al. conducted a randomized trial and found that the ultrasound group received surfactant earlier (1 h of life vs. 6 h, *p* < 0.001), with lower FiO_2_ (25 vs. 30%, *p* = 0.016) and lower CO_2_ (48 vs. 54, *p* = 0.011) (15). After surfactant treatment, newborns in the ultrasound group presented a greater SpO_2_ and SpO_2_/FiO_2_ ratio. LUSs lead to reduced oxygen exposure early in life and better oxygenation after treatment. Gregorio-Hernández et al. conducted a prospective study with newborns <35 weeks who needed noninvasive ventilation at birth (16). LUSs in the surfactant group were significantly higher than those in the no surfactant group. The ROC curve for surfactant treatment showed an AUC of 0.97 (95% CI 0.92–1). Vardar et al. conducted a prospective double-blind study (17). Neonates <34 weeks with clinical and radiological signs of RDS were evaluated with a six-area LUS examination in the first 2 h of life for the need for surfactant therapy. LUSs showed a significant correlation with the need for total surfactant doses. A cutoff LUSs of 4 predicted the need for surfactant with 96% sensitivity and 100% specificity (AUC: 1.00; 95% CI: 0.97–1.00). A meta-analysis also confirmed the accuracy of LUSs to guide surfactant replacement, and infants with LUSs >5–6 were at significantly increased risk of surfactant treatment compared with infants with LUSs <5–6 (37). Another systematic review is underway to test the accuracy of LUSs in the first day of life to predict surfactant treatment in preterm neonates (38).

These studies are paving the way for new applications of LUS, not only as an imaging technique but also as a functional tool. According to the above researches, from the perspective of zoning, the majority of studies conformed to the strategy of Brat et al. (Table 1) (exclusively scan the anterior and lateral chest in the supine position). Only one study adopts the 12-area strategy, including the posterior sections. And the result did not show more advantages (13). From the perspective of scoring, the majority of studies still follow the method proposed by Brat et al. (3). Only three of them use different scoring methods while they are still based on Brat et al. (3). Therefore, for neonatal RDS, the value of this scoring strategy has been confirmed.

## The LUSs in Neonatal BPD

BPD is one of the most common complications of prematurity affecting lung function and quality of life, accompanied by neurodevelopmental injury and retinopathy of prematurity (39, 40). Despite significant advances in neonatal care, the incidence of BPD has not decreased over the past decade (39). Researchers reported that new BPD is a chronic lung disease in surfactant-treated extremely low birth weight infants due to disruption of lung development with decreased septation, alveolar hypoplasia, and dysregulated development of pulmonary vasculature (41). BPD is diagnosed at 36 weeks of postmenstrual age (PMA), and a window of opportunity to provide some early management strategies is often missed. Some researchers have proposed that this window of opportunity encompasses the first 7–15 days of postnatal life, a concept that was supported by data from recent trials (42). According to data from the Neonatal Research Network, the prediction of BPD in the first 3 days of life (DOL) is mostly determined by GA, and from the 7th−28th DOL, the influence of other factors reflecting postnatal lung injury, such as ventilator support, increases (43). Hence, finding early biomarkers of developing BPD is needed to stratify individual risk soon after birth and implement preventive and therapeutic strategies when they can still alter the pathologic process (44). Detecting early markers of BPD is challenging, and biochemical, clinical, and radiological markers are currently being investigated, but most have either not shown sufficient accuracy or are not available in daily practice (45–48). Recent studies confirm that LUS has been shown to be very sensitive in assessing lung aeration in different settings (49). Loss of lung aeration early in postnatal life could be a biomarker of developing BPD.

LUS is increasingly recognized as a useful tool in preterm infants with BPD. Many studies have proved that LUSs in the early days after birth is a predictor of BPD (18–24, 50). A prospective study in infants with GA <34 weeks showed that LUSs in the first day of life did not predict the development of BPD (17). In another prospective study by Alonso-Ojembarrena et al. (18) 59 very low birth weight infants and/or GA ≤ 32 weeks were included. Six lung zones (upper anterior, lower anterior, and lateral; posterior lung zones were not evaluated) were scanned, and lung aeration was classified using the scoring system as the preceding study (3). LUS was performed on the 1st and 3rd DOL; postnatal weeks 1, 2, 3, and 4; and 36 weeks of PMA. They found higher LUSs from 1 week to 36 PMA in infants who developed BPD of any grade. LUSs in the 1st and 2nd weeks were moderately and highly predictive of any grade BPD, respectively. In a retrospective study of 27 infants born <30 weeks of GA, Abdelmawala et al. performed eight-zone LUS at a median postnatal age at the time of LUS studies of 5 (2–8) weeks and found that a LUSs of 6 had a remarkable performance in predicting BPD (19). However, in this study, LUS was not performed at a predefined time point; it was spread over a wide interval of postnatal age, and the authors did not assess posterior lung zones. Similar to other inflammatory lung diseases, such as bronchiolitis and acute RDS, dependent lung zones are the most affected. In preterm infants who develop BPD, the posterior lung fields are generally less aerated (20). Some researchers have incorporated the scanning of posterior lung fields based on the assumption that assessment of the dependent distribution of lung aeration may add to the predictive capability of the LUSs (15). Aldecoa-Bilbao et al. performed LUS at admission, at 7th, and 28th DOL with a standardized protocol (6 zones: anterior, lateral, and posterior fields) in a prospective observational study (22). They found that mean LUSs were significantly higher in patients with BPD at 7th and 28th DOL than those without BPD. LUSs at 7th DOL showed an AUC = 0.87 (0.79–0.94), *p* < 0.001 to predict NICHD 2001-BPD with a cutoff point ≥8, an AUC = 0.80 (0.70–0.90), *p* < 0.001 to predict Jensen 2019-BPD with a cutoff point ≥9. In this line, a prospective study was conducted by Liu et al., (23) and three different protocols (the classical 6-region, 10-region, and 12-region) were adopted. LUS was performed on the 1st, 2nd, and 3rd DOL and then once every 3 days until the 15th DOL. Every echogram was analyzed and graded from 0–3 points as described by Brat et al. (3). The 6-region (upper anterior, lower anterior, and lateral), 10-region (upper anterior, lower anterior, lateral, and upper posterior and lower posterior), and 12-region (upper anterior, lower anterior, upper lateral, lower lateral, upper posterior, and lower posterior) scores were calculated. Researchers found that the 12-region and 10-region LUS scoring protocols were superior to the 6-region LUS scoring protocol. There was no statistically significant difference between the 10-region and 12-region protocols. The best LUS timing to predict the presence of BPD was from the 9th to 15th DOL. Similar results were also published by Gao et al. (50). A recent prospective study by Oulego-Erroz et al. (20) enrolled 42 infants with GA <32 weeks. The scanning protocol included the assessment of four lung zones in each lung (upper anterior, lower anterolateral, lower posterolateral, and lower posterior). A LUSs was calculated on the 7th DOL and repeated on the 28th DOL. The results showed that infants in the moderate–severe BPD group (sBPD) had higher LUSs on the 7th and 28th DOL than infants in the non-sBPD group. The LUSs on the 7th DOL had an AUC of 0.94 (95% CI 0.87–1) for the diagnosis of sBPD at 36 weeks of PMA (optimal cutoff of ≥8 points: sensitivity 93%, specificity 91%). The assessment of posterior lung zones appeared to be important to improve diagnostic accuracy. However, late LUS had a worse predictive value for sBPD diagnosis than early LUS. Similarly, Loi B et al. conducted a multicenter study, and 147 neonates were included with GA <31 weeks (21). LUS was performed on the 1st, 7th, 14th, 28th DOL, and at 36 weeks PMA. LUS was scanned over 6 chest areas (three per side). LUSs were calculated and correlated with simultaneous blood gases and work of breathing score. The results showed that LUSs significantly correlated with oxygenation indicators and work of breathing at any time point. GA-adjusted LUSs significantly predicted BPD on the 7th and 14th DOL. Another multicenter study scanned the mid-clavicular, anterior, and posterior axillary lines of both hemithoraces (10). The results showed that in infants 25 Gao 30 weeks GA, the LUS at 7 DOL predicted BPD with an AUC of 0.82 (95% CI: 0.71 Gao 93). The 25 Gao 27 week group had an AUC of 0.5, whereas the 28 Gao 30 week group had an AUC of 0.89, which suggested an interaction between LUS and GA. In a recent multicenter study with 298 infants born before 32 weeks of GA (24), Alonso-Ojembarrena et al. adopted two LUSs protocols, one involving anterolateral lung fields (LUSs-al) and the other adding posterior fields (LUSs-p) at birth, the 3rd DOL, the 7th DOL, the 14th DOL, and the 21st DOL. The results suggested that both LUSs-p and LUSs-al showed a similar moderate diagnostic accuracy to predict msBPD on the 3rd DOL, 7th DOL, and 21st DOL. The LUSs-p was slightly more accurate at 14th DOL. A recent meta-analysis of seven studies showed that LUSs could accurately predict BPD and moderate-to-severe BPD at 7 and 14 days of life in preterm infants of gestational age <32 weeks. In addition, the diagnostic accuracy of LUSs and extended LUSs did not differ at any timepoint (51).

Six studies adopt the extended scanning strategy, including the posterior sections [42 Gao 47]. Three of the studies compared classic LUSs with extended LUSs, and there exists no conclusive evidence that extended LUSs are superior to classic LUSs in terms of diagnostic accuracy (22, 50, 52). This must be further investigated in future studies. Differences in the LUS predictive power between investigations may be attributable to the study design, population, and LUS scanning protocol. LUS has a significant correlation with GA, and the initial LUS was significantly higher in less mature infants (3). Above all, these findings suggest that the LUS may be useful as an early marker of BPD with the advantages of being safe and easy to perform, non-invasive, not painful, and not involving ionizing radiation.

## The LUSs in Neonatal Respiratory Support

LUS has been correlated with multiple indices of oxygenation and lung injury, and high LUSs are accurate at predicting the need for respiratory support in term and preterm neonates (3, 11, 52–55). Although CXR is widely used traditionally, it has a poor correlation with lung function, (52) and when a direct comparison has been attempted, CXR was often found to perform worse than LUS (9, 53).

In an original study by Raimondi et al., LUS could predict the need for respiratory support in neonates; however, they used a proposed LUS pattern grade, which they described as not semi-quantitative LUSs (52). Later, the author conducted a study that enrolled 54 infants. After a 2-h nasal ventilation trial, LUS could predict the need for intubation, largely outperforming conventional radiology, and the bilateral type 1 lung profile had a sensitivity of 88.9%, specifically 100% (35). Accordingly, Pang et al. divided each lung into six areas (upper and lower areas of anterior, posterior, and lateral sections). For each lung area, a 0–3 point score was given (13). The author found that LUSs for predicting mechanical ventilation (MV) showed 81.3% sensitivity and 88.8% specificity using a cutoff of 25.5 (AUC = 0.912; *P* < 0.001). Later, Szymański et al. proposed modified LUSs in neonates, which include posterior instead of lateral lung fields, and a 5-grade rating scale instead of a 4-grade rating scale (25). Seventy preterm infants <32 weeks GA and birth weight <1,500 g were scanned. Assessments were performed within 24 h of birth (LUS 0) and on Days 2, 3, 5, 7, 10, 14, 21, and 28. The results suggested that LUSs significantly correlated with SpO_2_/FiO_2_ (Spearman rho =- 0.635; *p* < 0.0001). Significant predictors of ventilation requirements on DOL 3 were LUS 0 (*p* < 0.016) and birth weight (*p* < 0.001). LUS 0 had high reliability in prognosing invasive ventilation on DOL 3 (AUC = 0.845; 95% CI: 0.738–0.951; *p* < 0.001) (25). Abushady et al. also found that patients who underwent LUSs guided recruitment maneuver achieved earlier lowest FiO_2_, shorter O_2_ dependency, shortening the duration of invasive ventilation, and marked decrease in lung inflammation (56). A prospective double-blind study was conducted in infants with a GA <34 weeks with RDS by evaluation with LUS and CXR on admission (17). A significant correlation was observed between high LUSs shortly after birth and PEEP levels. A significantly higher LUSs was observed in patients with CPAP failure. LUSs accurately predicted CPAP failure (AUC = 0.804; 95% CI: 0.673–0.935; *p* = 0.001). However, there was no correlation observed between LUSs and CPAP days.

Invasive ventilation is a lifesaving solution for critically ill neonates. Prolonged MV is associated with increased pulmonary complications, mortality, morbidity, and neurodevelopmental disability in neonates (57, 58). Limiting the duration of invasive ventilation and early weaning is important for minimizing these complications. However, premature infant weaning is associated with extubation failure (EF), which can cause poor outcomes (59). EF is common in the NICU and approximately 24–42% of neonates in previous studies (59–61). A prolonged duration of MV was also recognized as a risk factor for EF (62). Until now, the process of weaning from MV has remained a challenge and inexact (59–61). Therefore, choosing the optimal time for weaning and predicting EF is of great clinical significance. LUS was reported to predict weaning success and post-extubation failure in several studies in adults (63, 64). In addition, few studies have been carried out in neonates. The loss of pulmonary aeration following extubation can predict EF, as it represents loss of lung volume for gas exchange (65). Lung aeration loss can be evaluated by LUSs (66). LUS enables a dynamic assessment of lung aeration changes, unlike CXR. In a prospective study including 40 neonates with different causes of RD needing MV regardless of their GA. LUS was performed at least three times, at admission, before switching MV mode, and before weaning. Six areas per hemithorax (anterior, lateral, and posterior; each area was divided into two, superior and inferior) were scanned. Patients successfully weaned from SIMV showed significantly lower scores than those who failed. ROC analysis reported that LUS showed a sensitivity of 87.5% and a specificity of 100% to predict weaning success at a score of six (26). In a recent prospective trial, El Amrousy et al. assessed three chest areas for each lung: the upper anterior, the lower anterior and the lateral, with a total of six areas in both lungs. This study included 80 consecutive neonates on MV suffering from different pulmonary diseases. All patients underwent LUS just before extubation and 6 h after extubation. In this study, LUSs before and after extubation were significantly higher in neonates with EF than in those with weaning success. Post-extubation LUS had a sensitivity of 89% and a specificity of 90% to predict weaning success in neonates at a cutoff point ≤ 6 (27). The results for LUS agreed with previously reported results in adults (66, 67). Moreover, neonates with EF had significantly lower GA and lower weight compared to those with succeeded extubation. This was consistent with the results of other investigators (60, 68).

## Other Applications of LUSs

In addition to the applications above, a prospective study showed that using three-point LUS can predict admission to the NICU for TTN or RDS in term- and late-preterm infants (69). A 12-region scan protocol was adopted to assess the process of lung liquid clearance during the first 24 h. The LUSs at 6 h were significantly lower than those at <3 h, and within 3 h, B-lines were more abundant in the posterior chest and lower chest (70). Alonso-Ojembarrena et al. detected that diuretic responders showed lower LUSs, and that respiratory support decreased after diuretics in preterm infants before 32 weeks (71). Zhao et al. investigated LUSs in the assessment of pulmonary edema in low-weight neonates with patent ductus arteriosus (PDA) and found a significant difference in LUSs and aortic root ratio to left atrium (AO/LA) (72). Yu et al. assessed the lung water content by LUSs in very low-weight preterm neonates with persistent PDA. The LUSs and LA/AO ratio in the PDA group were higher than those in the control group, and the ROC results showed that LUSs had moderate accuracy for predicting hemodynamic changes in PDA (AUC =0.741; 95% CI: 0.621–0.839) (73). After congenital cardiac surgery, Kaskinen et al. showed that LUSs in the assessment of postoperative extravascular lung water can predict the length of MV and ICU stay, and it had less interobserver variability than CXR (74). Similarly, a prospective study by Girona-Alarcón et al. (75) showed a significant correlation between higher lung ultrasonography in cardiac surgery (LUCAS) score prior to surgery and longer MV, and high LUCAS score after surgery correlated with longer cardiopulmonary bypass time, inotropic support, and FiO_2_ need. In addition, LUSs significantly correlated with histological injury score and with several inflammatory markers (54, 76). Recent research has even demonstrated that LUSs is significantly higher in COVID-19 newborns than in controls (77).

## Conclusion

Above all, LUSs has been widely applied in various scenarios in neonates. As a semi-quantitative evaluation method, LUSs can predict clinical intervention at an early stage and show advantages over conventional examination. In spite of slight variations in the used scoring systems, the results are all concordant and present the same conclusions regarding the use of LUSs.

## Author Contributions

All those listed authors contributed to the preparation of the manuscript. The preliminary draft of the review was prepared by HZ, BL, YF, YL, PH, and HS. JZ, ZH, and CY critically revised the draft manuscript. All authors read and approved the final manuscript.

## Funding

This work was Supported by Shenzhen Fund for Guangdong Provincial Highlevel Clinical Key Specialties (No. SZGSP009) and Sanming Project of Medicine in Shenzhen (SZSM201612045).

## Conflict of Interest

The authors declare that the research was conducted in the absence of any commercial or financial relationships that could be construed as a potential conflict of interest.

## Publisher's Note

All claims expressed in this article are solely those of the authors and do not necessarily represent those of their affiliated organizations, or those of the publisher, the editors and the reviewers. Any product that may be evaluated in this article, or claim that may be made by its manufacturer, is not guaranteed or endorsed by the publisher.
